# Cost-effectiveness analysis of combining traditional Chinese medicine in the treatment of hypertension: compound Apocynum tablets combined with Nifedipine sustained-release tablets vs Nifedipine sustained-release tablets alone

**DOI:** 10.1186/s12906-020-03091-3

**Published:** 2020-11-05

**Authors:** Qian Xu, Nan Yang, Shuang Feng, Jianfei Guo, Qi-bing Liu, Ming Hu

**Affiliations:** 1 West China School of Pharmacy Sichuan University, 17, Renmin South Road, 3rd Section, Chengdu, 610041 Sichuan China; 2 Division of Pharmacy Practice and Administrative Sciences, College of Pharmacy, University of Cincinnati Medical Center, Cincinnati, OH USA; 3 Department of Pharmacology, School of Pharmaceutical Science, Hainan Medical University, Haikou, Hainan China

**Keywords:** TCMs, Compound Apocynum tablets, Nifedipine sustained-release tablets, Blood pressure variability, Markov model, Cost-effectiveness analysis

## Abstract

**Background:**

We evaluated the long-term cost-effectiveness of antihypertensive traditional Chinese medicines (TCMs) and to compare the cost-effectiveness of a combined treatment consisting of compound Apocynum tablets and Nifedipine sustained-release tablets with the cost-effectiveness of treatment with Nifedipine sustained-release tablets alone.

**Methods:**

A Markov model was used to simulate the potential incremental cost-effectiveness per quality-adjusted life year (QALY) to be gained from compound Apocynum tablets and Nifedipine sustained-release tablets compared with Nifedipine sustained-release tablets alone. Model parameter estimates were informed by previously published studies. The direct medical costs of outpatients with hypertension were estimated from the health care provider’s perspective. A 5% annual discount rate was applied to both costs and QALYs.

**Results:**

TCMs combined with Nifedipine sustained-release tablets group generated a total 20-year cost of 11,517.94 RMB (US $1739.87), whereas Nifedipine sustained-release tablets alone group resulted in a 20-year cost of 7253.71 RMB (US $1095.73). TCMs combined with Nifedipine sustained-release tablets group resulted in a generation of 12.69 QALYs, whereas Nifedipine sustained-release tablets alone group resulted in 12.50. The incremental cost-utility ratio was 22,443.32 RMB (US $3390.23) per QALY. Considering the threshold of 1 GDP per capita in China in 2018 (US $9764.95), the combination of compound Apocynum tablets and Nifedipine sustained-release tablets was a cost-effective strategy. One-way and probabilistic sensitivity analysis showed unchanged results over an acceptable range.

**Conclusions:**

Combining Traditional Chinese Medicines with chemical medicines is more cost-effective strategy in the treatment of hypertension.

## Background

Hypertension is one of the most common chronic diseases that threatens human health, and it is also the major risk factor for cardiovascular diseases [1]. It has been reported [2] that 2 million people die of hypertension every year, 71% of the deaths caused by stroke are related to hypertension, and 53% of the deaths caused by coronary atherosclerotic heart disease are related to hypertension in China. Good blood pressure control is expected to avoid 350,000 to 600,000 deaths between 2016 and 2030 [3]. In 2015, the per capita medical expenses (outpatient and hospitalization expenses) of hypertension in China were $1123.88 per year, and the per capita medical expenses (including outpatient and hospitalization expenses) of patients with four primary hypertension complications were $1587.16 per year [4].

With the progress of disease recognition, the goal of hypertension treatment has gradually changed from controlling blood pressure to improving complications related to target organ injury and improving blood pressure variability to reduce the long-term risk of cardiovascular (CVD) [5]. Yikona et al. found that an increase in blood pressure variability (BPV) is related to the occurrence, development and severity of cardiovascular and renal injury [6]. An increase in BPV in 24 h was related to the incidence and mortality of cardiovascular events [7, 8]. Standard deviations (SDs) and coefficient of variations (CVs) are often used as indicators of BPV in the clinic [9]. The results of a post-analysis of a large-scale intervention trial for hypertension in 2010 showed that BPV measured during the consultation period had a strong predictive value for cardiovascular morbidity [9]. Even in some cases, the correlation between BPV and cardiovascular incidence is stronger than the relationship between mean blood pressure and BPV [10].

Previous studies have found that traditional Chinese medicines (TCMs) not only have an obvious effect on the improvement of BPV, with the characteristics of stabilize blood pressure and better control of 24-h ambulatory blood pressure but also reduce adverse events and protect target organs, including the heart, brain and kidney [11–15]. Previous studies indicated that TCMs (e.g., the liuwei dihuang pill and tianma gouteng yin) were effective and safe for primary hypertension when compared with conventional treatments (e.g., diuretics, beta-blockers, calcium-channel blockers, and ACE inhibitors) [16, 17]. According to the *Guidelines for the Rational Use of Hypertensive Drugs in China and the Chinese expert consensus document on antihypertensive therapy with single-pill combination* [18, 19], it is indicated that two or more drugs should be used together. Chemical antihypertensive drugs combined with traditional Chinese medicine with different antihypertensive mechanisms can achieve blood pressure and reduce adverse events.

Compound Apocynum tablets are composed of Apocynum, Stephania tetrandra, *Chrysanthemum indicum*, etc. [20]. Some studies have shown that Apocynum extract can reduce blood pressure by enhancing the production and release of nitric oxide [21] or improving renal function. In addition, Compound Apocynum extract can be used for the treatment of hypertension, and it protects liver and decreases anxiety and depression [21, 22].

Most of the efficacy, safety and economic evaluations of antihypertensive drugs used systolic blood pressure (SBP) or diastolic blood pressure (DBP) as the main clinical indicator, and there is less research in which blood pressure variability is the main indicator. Therefore, we tried to construct a Markov model based on 24-h ambulatory blood pressure monitoring to simulate the disease development process of hypertension in living patients who were without stroke/myocardial infarction (MI), stroke, or MI. The long-term cost effectiveness of blood pressure reduction using Nifedipine sustained-release tablets combined with compound Apocynum tablets was further explored and compared with Nifedipine sustained-release tablets alone to provide evidence for the long-term economic effect of antihypertensive Chinese patent medicines on improving BPV.

## Methods

### Model description and structure

**Fig. 1:**
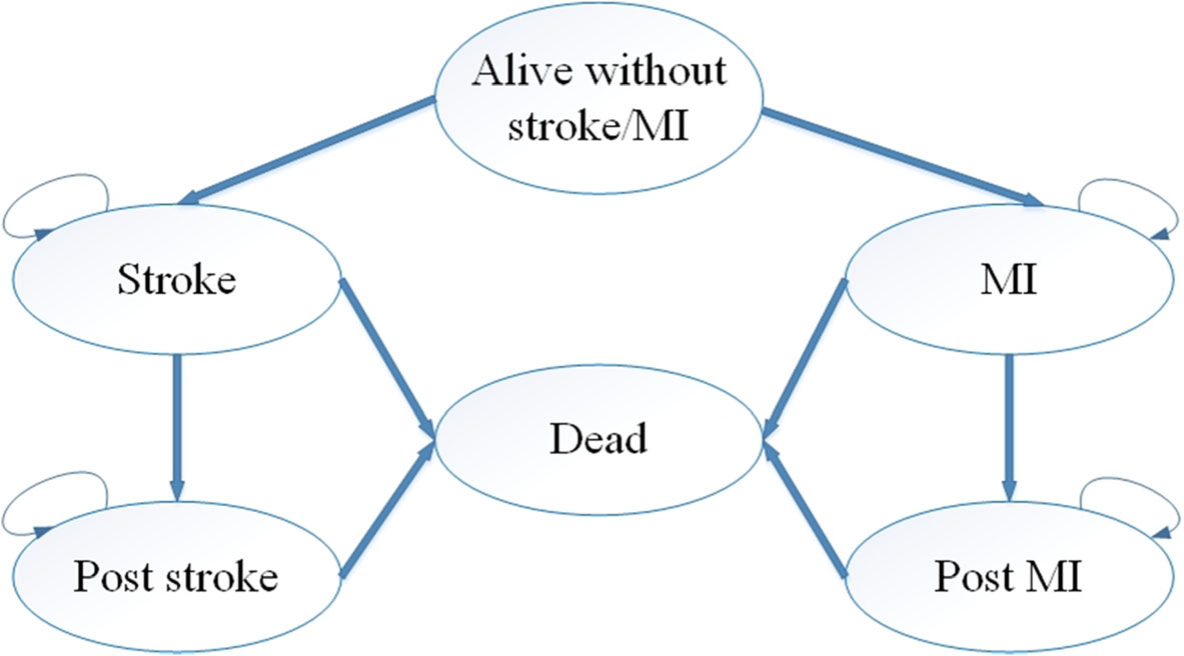
Bubble Map of the Markov Model in Hypertensive Patients

We developed a Markov model, using TreeAge Pro 2011 (TreeAge Software, Williamstown, MA, USA), from a Health care provider’s perspective to compare 20-year timeframe costs and health benefits associated with compound Apocynum tablets and Nifedipine sustained-release tablets for patients with hypertension. Considering the long-term effects of BPV on patients with hypertension, the development and prognosis of hypertension and the literature, our Markov model was developed for six health states: alive without stroke/ MI,MI, stroke, MI, post-stroke, post-MI and dead. In addition, death was the absorbing state. We assumed that patients could not be in more than one state at the same time, which conformed to Markov’s health states setting rules. Because hypertension is a chronic disease, its related complications take many years to manifest. Therefore, the cycle length was set to 1 year, with a time horizon of 20 years. The state transition bubble model is as follows (Fig. 1).

Considering the availability of data, our model makes the following assumptions. 1) All patients are alive without stroke/MI state when they enter the model; 2) There is a linear relationship between the incidence of the base case and time; 3) The BPV index does not change linearly with time; and 4) The cost and utility of the absorbing state was 0. We took 1 time the GDP per capita ($9764.95) as the cost-effectiveness analysis threshold [23–26], and the discount rate was 5%.

### Parameter input

#### Transition probabilities

In this study, we tried to use blood pressure variability to calculate the transfer probability required by the Markov model. Studies showed in the prediction of cardiovascular disease and adverse events, nocturnal blood pressure showed better prognostic value than daytime blood pressure and 24-h blood pressure, because nocturnal blood pressure was less stimulated by physical activity, emotional stress, and the presence of the environment during the day [27, 28]. So nocturnal blood pressure was involved as the main clinical outcomes.

Our model parameters were extracted and calculated based on published literatures (Table 1). Literature searching of the effectiveness of TCM was conducted with inclusion criteria as following: (1) patients with hypertension, (2) treatments of TCM (or combined with TCM) compared with chemical medicines, (3) using BPV as primary or secondary effectiveness indicator, (4) Randomized controlled trials. No BPV indicator, non-RCT, studies were excluded. We used “hypertension”, “BPV”, “TCM”, RCT and other keywords to search MEDLINE (via OVID), EMBASE (via OVID), CNKI, Wan Fang, and VIP for articles published before March 2018.

**Table 1:** Markov simulation parameters for BPV

Items	Base Case	Range	Source
Nocturnal Blood Pressure ΔSD			
Nifedipine	1.87	1.683 ~ 2.057	[29]
Compound Apocynum+Nifedipine	2.99	2.691 ~ 3.289	[30]
Nocturnal Blood Pressure ΔCV			
Nifedipine	0.00		[29]
Compound Apocynum+Nifedipine	0.01	0.009 ~ 0.011	[29]
Baseline incidence of stroke for 1 year	1.1%	0.01 ~ 0.012	[31]
Incremental stroke incidence for 1 year	0.320%	0.003 ~ 0.004	[30]
Incidence of stroke			
Nifedipine	0.460%	0.0041 ~ 0.0051	
Compound Apocynum+Nifedipine	0.102%	0.0009 ~ 0.0011	
Baseline incidence of MI for 1 year	0.2%	0.0018 ~ 0.0022	[31]
Incremental MI incidence for 1 year	0.811%	0.0073 ~ 0.0089	[30]
Incidence of MI			
Nifedipine	0.199%	0.0018 ~ 0.0022	
Compound Apocynum+ Nifedipine	0.193%	0.0017 ~ 0.0021	

Finally, we found only one RCT study. In this study, 108 patients were included in. They were divided into two groups, nifedipine sustained-release tablets (10 mg, qd) and nifedipine sustained-release tablets (10 mg, qd) combined with compound Apocynum tablets (2 tablets, tid) were given for 4 weeks respectively for the treatment of primary hypertension patients, and there was no difference in baseline between the two groups [29]. 24-h ambulatory blood pressure monitor were used for blood pressure monitoring before and after treatment. The results showed that nifedipine sustained-release tablets combined with compound Apocynum tablets could reduce blood pressure and the variability of blood pressure meanwhile. The Standard Deviation (SD) of systolic blood pressure was 2.99 mmHg, which could reduce the Coefficient of Variation (CV) of systolic blood pressure by 0.01; nifedipine sustained-release tablet group could reduce the SD by 1.87 mmHg, the CV of systolic blood pressure remained unchanged.

For the incidence of related events, we searched published RCTs, cohort studies, epidemiological literature of disease population, reviews, and national health statistical annual reports, and related statistical data, using key words “myocardial infarction”, “stroke”,“death”,“the incidence of disease”and so on. The increase in the incidence of related events caused by the change of related indicators came from the study of Tai et al. [30], which found that for every 1 mmHg increase in the standard deviation of systolic blood pressure (SD), the incidence of stroke would increase by 2%. For every 1% increase in CV of SBP, the risk of MI would increase by 5%. The incidence of basic events was derived from a 5.5-year follow-up of 457 patients with hypertension in Japan. The results showed that 26 patients (5.69%) had stroke and 5 patients (1.09%) had MI. The 1-year incidence was calculated according to the probability formula [31].

The transition probabilities of stroke and MI in a single cycle of Nifedipine sustained-release tablets and Nifedipine sustained-release tablets combined with compound Apocynum tablets were calculated by multiplying the decrease in the SD of blood pressure and the CV of a certain drug with the increase in incidence of related events caused by the change of relevant indicators and the incidence of basic events.

The probability of recurrence of and death from stroke came from a 5-year follow-up study in Singapore [32]. The results showed that the rates of recurrence and metastasis for stroke patients were 41.70% and 13.03%, respectively. Data on the incidence of post-stroke and death were from Chang et al. [33]. The probability of recurrence and death after MI came from Canada’s 1-year follow-up of 8493 patients with MI. The results showed that the incidence rates were 12.50% and 9.70% for recurrence and death, respectively [34]. The probability of death Post-MI comes from the study of Chiang et al. [35]. When the research time was not consistent with the time of a single cycle length, it was converted by the following probability transfer formula.

$$ \mathrm{Transition}\ \mathrm{probability}\ \mathrm{formula}:{\mathrm{t}}_{\mathrm{P}}=1-\left(1-\mathrm{P}\right)1/\mathrm{T} $$

t: a cycle length; t_P_: transition probability per cycle length; P: event rate; T: time horizon.

#### Utility and cost

We used “hypertension”, “myocardial infarction”, “stroke”, “post-stroke”, “post myocardial infarction”,“health utility” as our keywords and searched PubMed to get the health utility of different states [36, 37]. The medicines cost of “Compound Apocynum” and “Nifedipine” collected from the YAOZHI website [38]. Cost of hospitalization for patients in different states collected from *The Health Statistical Yearbook of China in 2013* [39] and a study by Chan et al. [40]. In this study the cost was calculated on a yearly basis (Table 2). All searches were by March 2018.

**Table 2:** Summary of inputs used in the base-case model

Items	Base Case	Range	Source
Baseline age	67.5	60 ~ 75	MF Ju [29]
**Transition probability**			
Alive without stroke/MI **→** stroke			
Nifedipine	0.460%	0.0041 ~ 0.0051 0.0009 ~ 0.0011	[29–31]
Compound Apocynum+ Nifedipine Levamlodipine	0.102%	0.0009 ~ 0.0011	[29–31]
Alive without stroke/MI **→** stroke			
Nifedipine	0.199%	0.0018 ~ 0.0022	[29–31]
Compound Apocynum+ Nifedipine Levamlodipine	0.193%	0.0017 ~ 0.0021	[29–31]
Stroke →Stroke	41.7%(5 year)	0.3753 ~ 0.4587	[32]
Post-stroke →Death	25.6%(4 year)	0.2304 ~ 0.2816	[33]
Stroke →Death	13.03%(5 year)	0.1172 ~ 0.1433	[33]
MI → MI	12.50%	0.1125 ~ 0.1375	[34]
Post-MI → Death	6.10%	0.0549 ~ 0.0671	[35]
MI → Death	9.70%	0.0873 ~ 0.1067	[34]
**Utility**			
Alive without stroke	0.98	0.882 ~ 1	[36]
Stroke	0.5	0.45 ~ 0.55	[37]
MI	0.70	0.63 ~ 0.77	[37]
Post-stroke	0.63	0.567 ~ 0.693	[37]
Post-MI	0.8	0.72 ~ 0.88	[37]
**Cost (USD/per year)**			
Nifedipine drug costs	16.31	14.68 ~ 17.95	[38]
Compound Apocynum+Nifedipine drug costs ii	102.42	92.18 ~ 112.66	[38]
MI (in hospital)	2538.01	2284.26 ~ 2791.87	[39]
Stroke (in hospital)	1843.86	1659.56 ~ 2028.35	[39]
Post-stroke	1692.15	1522.93 ~ 1861.36	[40]
Post-MI	20,141.84	1837.66 ~ 2246.03	[40]

### Sensitivity analysis

A one-way sensitivity analysis and a probability sensitivity analysis (PSA) were used to verify the robustness of the results. For the one-way sensitivity analysis several factors that had the greatest impact on the results were chosen, including cost, utility, transfer probability that fluctuated by 10% [41, 42] and discount rate that fluctuated by 1% ~ 8% [23]. Combined with the source literature for the model parameters, the distribution method of cost was Gamma, and the distribution method of utility and transfer probability was Beta.

## Results

### Base case analysis

The Markov model was used for a cost-effectiveness analysis of the two treatments for hypertension (Table 3) in TreeAge Pro 2011. The cumulative cost after 20 years was $1739.87, and the health output was 12.69 QALYs in the compound Apocynum+Nifedipine group; the cumulative cost after 20 years was $1095.73, and the health output was 12.50 QALYs in the Nifedipine group. The incremental cost-effectiveness analysis showed that the ICER of compound Apocynum tablets combined with Nifedipine sustained-release tablets was $3390.23 compared with Nifedipine sustained-release tablets, which was lower than the threshold (1 GDP per capita = $9764.95).

**Table 3:** Base case results from the cost-effectiveness analyses

Treatments	Cost (USD)	QALYs	CER	ICER
Compound Apocynum+Nifedipine	1739.87	12.69	137.11	3390.23
Nifedipine	1095.73	12.50	87.66	

### Sensitivity analysis

#### One-way sensitivity analysis

**Fig. 2:**
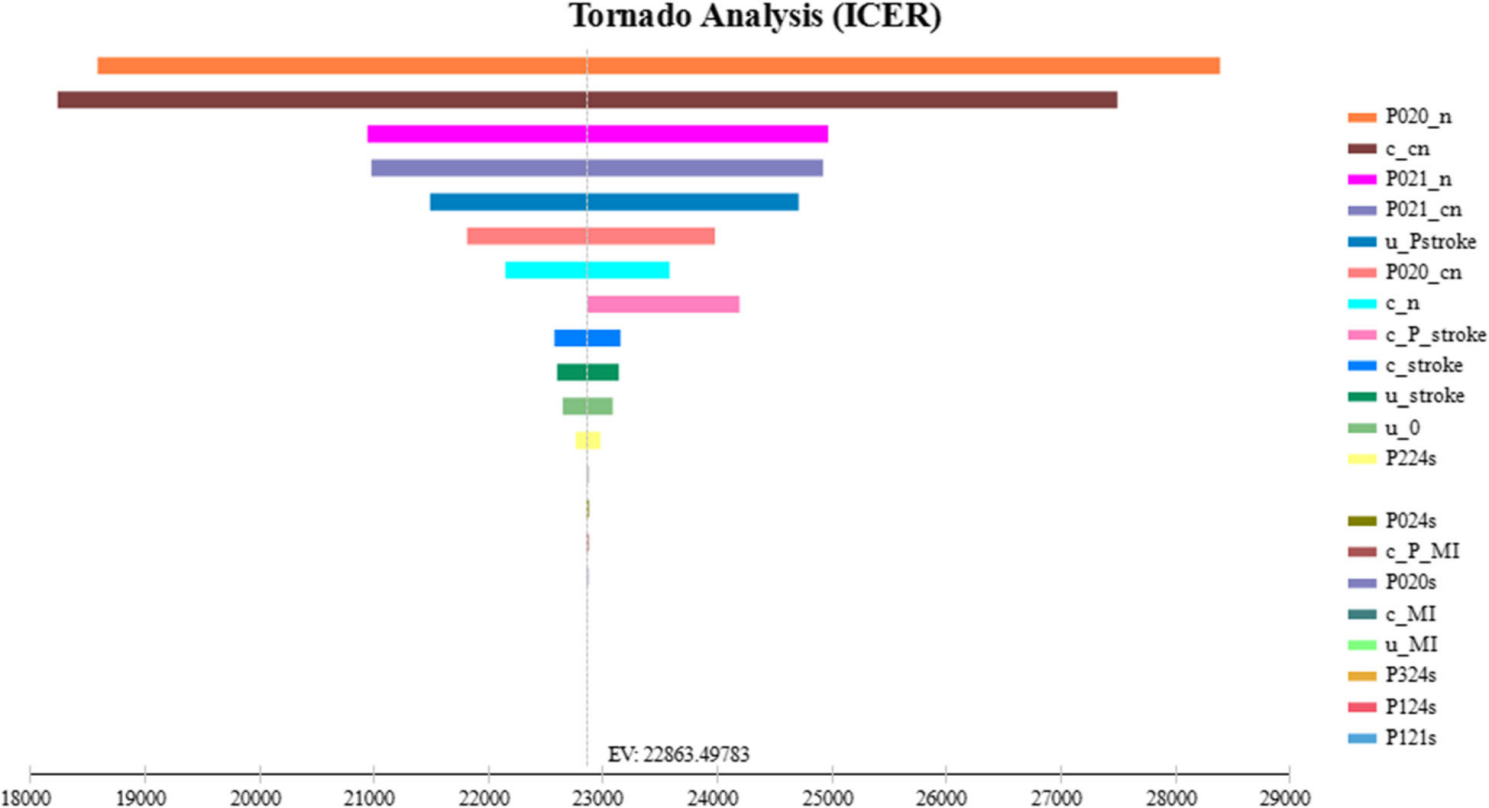
Tornado diagram from the one-way sensitivity analysis. (c_cn: Compound Apocynum+Nifedipine; c_P_MI: Cost of post-MI; c_MI: Cost of MI in hospital; c_stroke: Cost of stroke; c_n: Cost of Nifedipine; c_P_stroke: Cost of post-stroke; u_0: Alive without stroke/MI; u_PMI: Utility of post-MI; u_Pstroke: Utility of post-stroke; u_MI: Utility of MI;P021_cn: Transition probability from alive without stroke/MI to MI in the compound Apocynum+Nifedipine group; P020_cn: Transition probability from being alive without stroke/MI to stroke in the compound Apocynum+Nifedipine group; P224s: Transition probability from post-stroke to death; P124s: Transition probability from MI to death; u_stroke: Utility of stroke; P121s: Transition probability from MI to MI; P024s: Transition probability from stroke to death; P020s: Transition probability from stroke to stroke; P021_n: Transition probability from alive without stroke/MI to MI in Nifedipine; P020_n: Transition probability from alive without stroke/MI to stroke in Nifedipine; P324s: Transition probability from post-MI to death)

We carried out a one-way sensitivity analysis. A tornado diagram was developed to illustrate the sensitivity of ICER to changes in key parameters (Fig. 2). The five most sensitive parameters in the cost-effectiveness analysis were the transition probability from alive without stroke/MI to stroke in Nifedipine, the cost of compound Apocynum+Nifedipine, transition probability from alive without stroke/MI to MI in Nifedipine group, transition probability from alive without stroke/MI to MI in the compound Apocynum+Nifedipine group, and utility of post-stroke. The one-way sensitivity analyses of the top five factors have shown that the economic results are still stable, in other words, the ICER value is still at the threshold after the factor fluctuates by 10%. The one-way sensitivity analyses results were robust (Table 4).

**Table 4:** Results of the partial one-way sensitivity analyses

	Cost		QALY		CER		ICER	Results
	Compound Apocynum+Nifedipine	Nifedipine	Compound Apocynum+Nifedipine	Nifedipine	Compound Apocynum+Nifedipine	Nifedipine		
Base-case	1739.87	1095.73	12.69	12.50	137.11	87.66	3390.23	Dominant
P020_n increase10%	1739.87	1095.73	12.69	12.50	137.11	87.66	3390.23	Dominant
P020_n decrease 10%	1739.87	1095.73	12.94	12.75	134.46	85.94	3390.23	Dominant
C_cn increase 10%	1739.87	1095.73	11.41	11.26	152.49	97.31	4294.29	Dominant
C_cn decrease 10%	1870.81	1095.73	12.69	12.50	147.42	87.66	4079.39	Dominant
P021_n increase 10%	1608.93	1095.73	12.69	12.50	126.79	87.66	2701.07	Dominant
P021_n decrease 10%	1767.03	1095.73	12.68	12.50	139.36	87.66	3729.44	Dominant
P021_cn increase 10%	1712.65	1095.73	12.69	12.50	134.96	87.66	3246.96	Dominant
P021_cn decrease 10%	1739.87	1095.73	12.70	12.51	137.00	87.59	3390.23	Dominant
u_Pstorke increase 10%	1739.87	1095.73	12.68	12.49	137.21	87.73	3390.23	Dominant
u_Pstroke decrease 10%	1751.94	1095.73	12.68	12.50	138.17	87.66	3645.64	Dominant

(P020_n: Transition probability from alive without stroke/MI to stroke in Nifedipine; c_cn: Compound Apocynum+Nifedipine; P021_n: Transition probability from alive without stroke/MI to MI in Nifedipine; P021_cn: Transition probability from alive without stroke/MI to MI in the compound Apocynum+Nifedipine group; u_Pstroke: Utility of post-stroke.)

#### Probabilistic sensitivity analysis

**Fig. 3:**
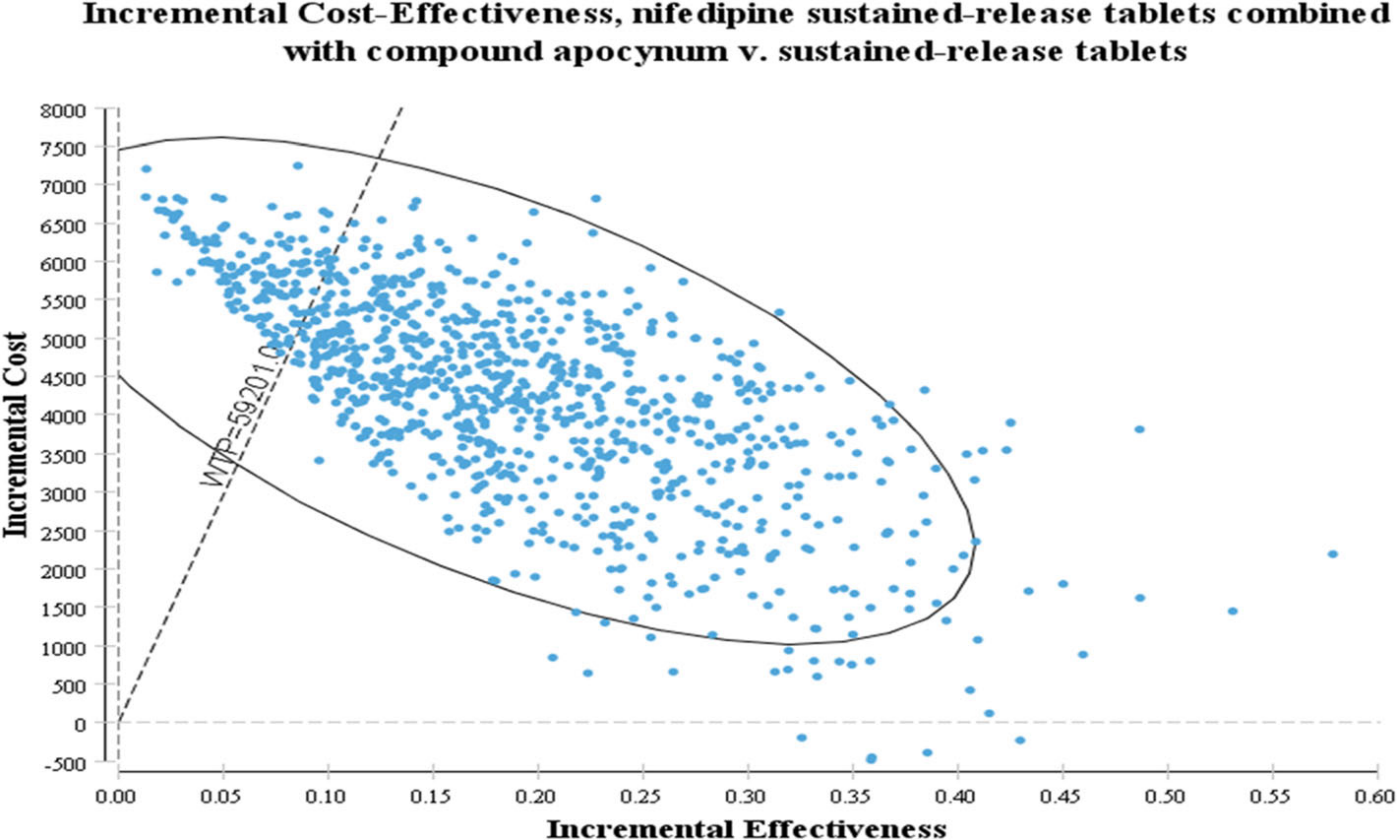
PSA Scatter plot of the incremental cost/QALY ratio

**Fig. 4:**
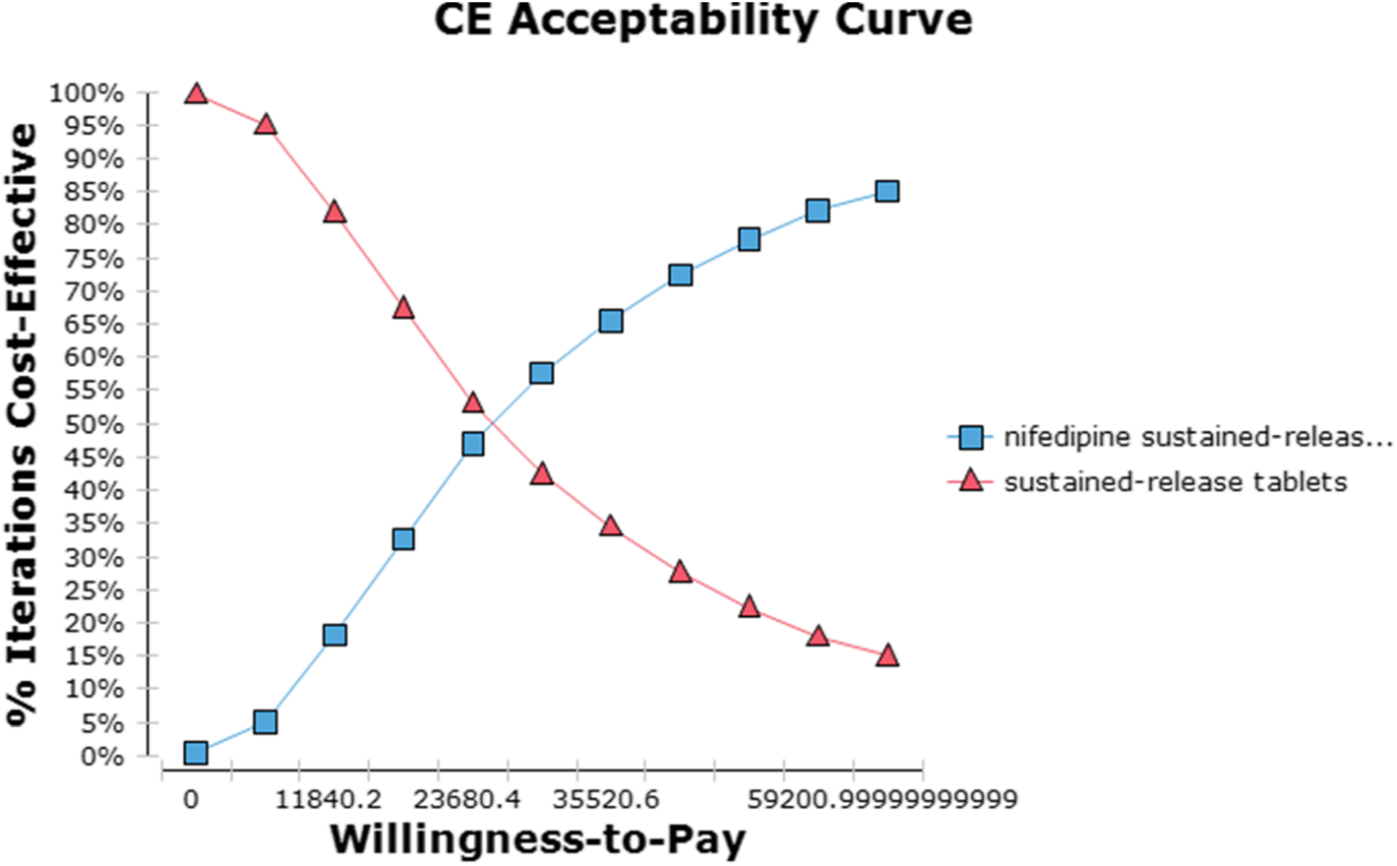
Cost-effectiveness Acceptability Curve for Compound Apocynum+Nifedipine vs Nifedipine. ( : Compound Apocynum+Nifedipine : Nifedipine)

The scatter plot of the PSA results (Fig. 3) shows that there was an 84.5% chance of the compound Apocynum+Nifedipine treatment strategy being cost-saving compared with Nifedipine. The shape of the scatter plot indicates a linear relationship between efficacy and cost; that is, the greater the number of QALYs obtained, the lower the incremental cost. A cost-effectiveness acceptability curve (CEAC) illustrates the probability that an intervention is more cost-effective compared with the alternative intervention(s). For different WTP thresholds, different strategies are optimal. With respect to WTP, as the value varied from $0 to $9764.95(59,201 RMB), the acceptable proportion of the compound Apocynum+Nifedipine group increased, while the acceptable percentage for the Nifedipine group decreased (Fig. 4).

## Discussion

Previous systematic review demonstrated that there is a relative lack of cost-effectiveness research in TCMs [43]. In this study, the Markov model was constructed based on BPV to simulate the long-term effects of TCMs on the health status of hypertensive patients. This study contains reference values for future long-term economic evaluations of antihypertensive TCMs. The evaluation results can also serve as a reference for the diagnosis and treatment of clinical primary hypertension patients.

The choice of research perspective in economic evaluation determines the measurement range of cost. This study selected the Health Care Provider’s Perspective. The results show that the combination of compound Apocynum tablets and Nifedipine sustained-release tablets was a cost-effective strategy in the Health Care Provider’s Perspective. In other words, it was economical to use TCMs combined with chemical drugs in Chinese medical institutions to treat hypertension patients. The different drug utilization patterns were impacted our results, such as combined with different Chemical antihypertensive drugs. However, pharmacoeconomic assessments were based on data from clinical trials or real-world data, and further TCMs clinical trials and real-world studies were recommended.

Although reducing hypertensive variability is an advantage of antihypertensive TCMs, there are still few clinical studies in China, and the existing studies had a lot limitations and were small in scale. Chen et al. [44] proved that the combination of Tianma Gouteng Decoction and amlodipine besylate tablets could reduce the 24-h average systolic and diastolic blood pressure of patients. Fang et al. [45] found that benazepril combined with antelope horn capsules could reduce morning blood pressure. Wang et al. [46] found that Songling Xuemaikang capsules combined with amlodipine besylate tablets could reduce the standard deviation of blood pressure in hypertension patients during the day and night, but the study lacked the corresponding data for mean blood pressure. Generally, most studies only describe the average blood pressure level in daytime, at night and over 24 h [47]. However, only one study [29] reported the SD and CV of blood pressure which are the most commonly used indicators of BPV. This study only compared the efficacy of chemical drugs alone with that of chemical drugs combined with TCMs. It did not compare a chemical drugs directly with a TCM used to reduce blood pressure. BPV affects the occurrence of stroke and MI. Some studies have found that BPV is closely related to kidney damage [11, 12]. When BPV increases, it may be due to an increase in microvascular resistance and small vessel resistance, which causes high blood pressure, high perfusion and high filtration. However, there is no data to quantify the relationship between BPV and renal damage, so this relationship has not been included in this study. Therefore, relevant empirical research to verify and improve the construction of this study is recommended to simulate long-term disease development in hypertensive patients.

This study had several limitations. First, in this study, the parameters such as efficacy and disease state transition probability were from the limited literature available at present. For example, the clinical efficacy data of two drug treatment schemes were from a small sample of short-term RCT, and the incidence of stroke caused by blood pressure variability was from the international literature rather than the Chinese population. All of these brought certain uncertainty to the calculation results of the model. And we needed more data from large samples of long-term RCT with blood pressure variability as an indicator and epidemiological studies based on the relationship between blood pressure variability and stroke, MI, etc. in Chinese population to update our study. Second, the health utility values were one of the key parameters in cost-effectiveness analysis, however, we didn’t found study about the utility values of Chinese population. Therefore, it is suggested to carry out utility measurement based on Chinese population in the future. Third, we did not fully explore other therapeutic strategies for TCMs combined with Antihypertensive chemical drugs. This paper has presented a long-term economic evaluation method of TCM combined with chemical drugs in the treatment of hypertension. Therefore, it was suggested to compare the economics of more treatment strategies in future studies.

## Conclusions

The pharmacoeconomic results for Nifedipine sustained-release tablets combined with compoundApocynum compared with Nifedipine sustained-release tablets alone indicated that the total cost of compound Apocynum+Nifedipine after 20 years was $1739.87 and the health output was 12.69 QALYs; the cumulative cost of Nifedipine for 20 cycles was $1095.73, and the health output was 12.50 QALYs. The results of the incremental cost-effectiveness analysis are presented. The ICER of compound Apocynum tablets and Nifedipine sustained-release tablets was lower than the threshold value, so it is economical.

## Data Availability

All data generated or analysed during this study are included in this published article [[1] Ju MF,Wang H, Hong R, et.al. Observation on curative effect of Compound Apocynum tablets in the treatment of senile essential hypertension [J]. J Chengde Med Coll. 2014(06): 532–533. [2] Tai C, Sun Y, Dai N, et al. Prognostic significance of visit-to-visit systolic blood pressure variability: a meta-analysis of 77,299 patients [J]. J Clin Hypertens (Greenwich). 2015, 17(2): 107–115. [3] Eguchi K, Hoshide S, Schwartz J E, et al. Visit-to-visit and ambulatory blood pressure variability as predictors of incident cardiovascular events in patients with hypertension [J]. Am J Hypertens. 2012, 25(9): 962–968. [4] Sun Y, Lee S H, Heng B H, et al. 5-year survival and rehospitalization due to stroke recurrence among patients with hemorrhagic or ischemic strokes in Singapore [J]. BMC Neurol. 2013, 13: 133. [5] Chang K C, Lee H C, Tseng M C, et al. Three-year survival after first-ever ischemic stroke is predicted by initial stroke severity: A hospital-based study [J]. Clin Neurol Neurosurg. 2010, 112(4): 296–301. [6] Tangri N, Ferguson T W, Whitlock R H, et al. Long term health outcomes in patients with a history of myocardial infarction: A population based cohort study [J]. PLoS One. 2017, 12(7): e180010. [7] Chiang F T, Shyu K G, Wu C J, et al. Predictors of 1-year outcomes in the Taiwan Acute Coronary Syndrome Full Spectrum Registry [J]. J Formos Med Assoc. 2014, 113(11): 794–802. [8] Stein J D, Brown G C, Brown M M, et al. The quality of life of patients with hypertension [J]. J Clin Hypertens (Greenwich). 2002, 4(3): 181–188. [9] Ara R, Tumur I, Pandor A, et al. Ezetimibe for the treatment of hypercholesterolaemia: a systematic review and economic evaluation [J]. Health Technol Assess. 2008, 12(21): 1–212.], some data were collected from YAOZHI website [https://www.yaozh.com/] and *The Health Statistical Yearbook of China in 2013.*

## Abbreviations

TCMs: Traditional Chinese medicines
QALYs: Quality-adjusted life-years
CVD: Cardiovascular
BPV: Blood pressure variability
SD: Standard deviations
CV: Coefficient of variations
SBP: Systolic blood pressure
DBP: Diastolic blood pressure
MI: Myocardial infarction
PSA: Probabilistic sensitivity analysis
WTP: Willingness to pay
